# Supplementary material for: Porphyromonas gingivalis HmuY and Bacteroides vulgatus Bvu—A Novel Competitive Heme Acquisition Strategy
Source: Int J Mol Sci. 2021 Feb 24;22(5):2237. doi: 10.3390/ijms22052237 (PMC7956564; doi:10.3390/ijms22052237)
Supplement: Supplementary file 1 [file ijms-22-02237-s001.pdf]

Porphyromonas gingivalis HmuY and *Bacteroides vulgatus* Bvu – novel competitive heme acquisition strategy in the gut microbiome

Klaudia Siemińska, Patryk Cierpisz, Michał Śmiga and Teresa Olczak*

[1] Laboratory of Medical Biology, Faculty of Biotechnology, University of Wrocław, 14A F. Joliot-Curie St., 50-383 Wrocław, Poland; klaudia.sieminska@uwr.edu.pl (K.S.); patryk.cierpisz2@uwr.edu.pl (P.C.); michal.smiga2@uwr.edu.pl (M.Ś.); teresa.olczak@uwr.edu.pl (T.O.)

* Correspondence: teresa.olczak@uwr.edu.pl

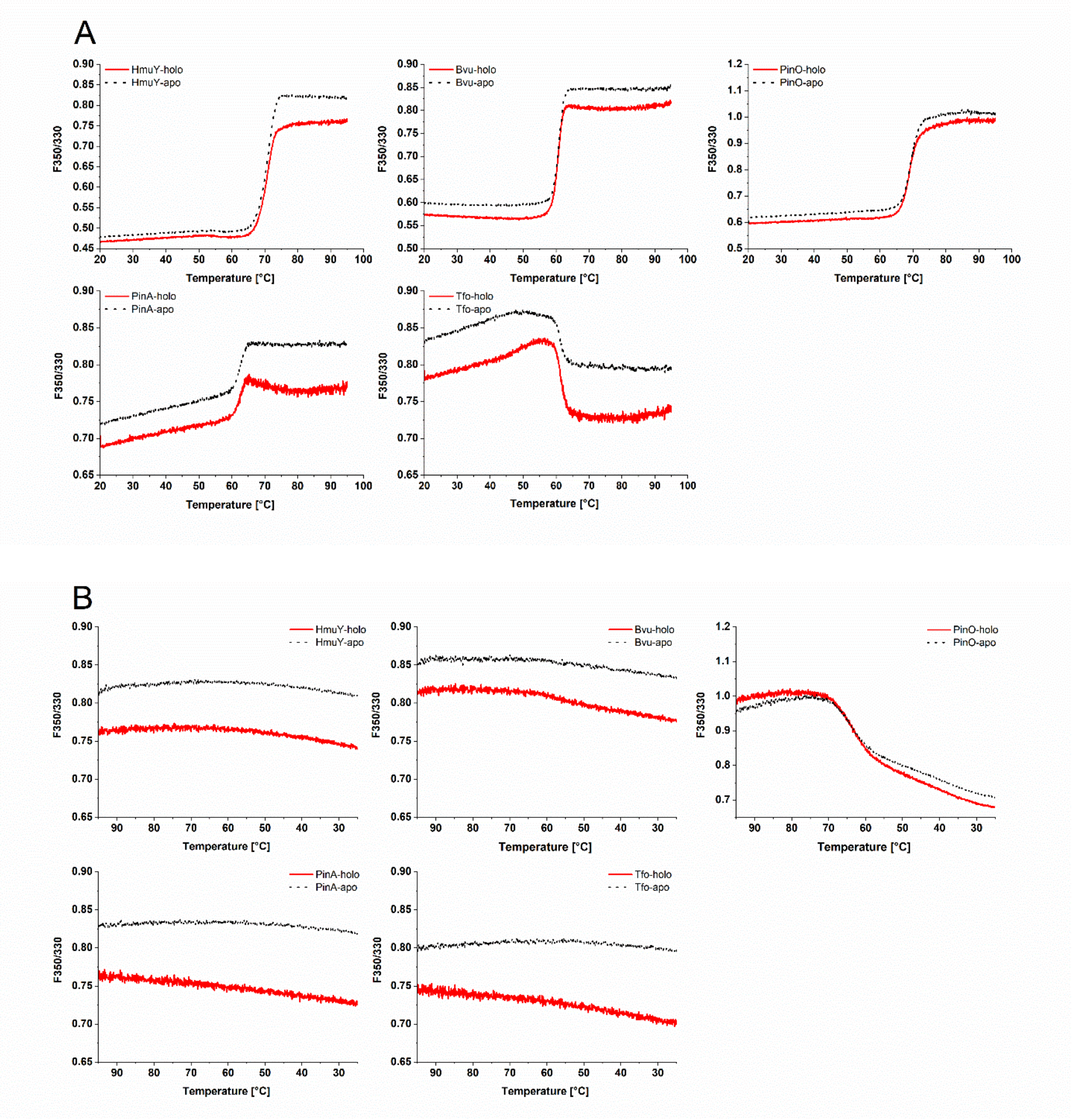

Figure S1. Conformational stability of hemophore-like proteins. Thermal unfolding (A) and refolding (B) of apo-proteins (red line) and holo-proteins complexed with heme (black line) was examined using a label-free fluorimetric analysis. Proteins examined: HmuY, *P. gingivalis*; Bvu, *B. vulgatus*; PinO, *P. intermedia*; PinA, *P. intermedia*; Tfo, *T. forsythia.*
